# Case Report: Major right-sided hepatectomies in infants in Uzbekistan: a case series

**DOI:** 10.3389/fped.2024.1495165

**Published:** 2025-01-21

**Authors:** K. Semash, T. Dzhanbekov, S. Islomov

**Affiliations:** National Children’s Medical Center, Tashkent, Uzbekistan

**Keywords:** hepatectomy, infant, liver neoplasms, hepatoblastoma, hemangioma, pediatrics, liver surgery

## Abstract

**Introduction:**

Liver resection is a critical surgical option for managing benign and malignant tumors, including rare and complex cases in pediatric patients. While such procedures are well-documented in adults, the surgical management of large liver neoplasms in infants poses unique challenges due to anatomical and physiological considerations, as well as the limited number of cases reported globally.

**Aim:**

This study aimed to describe the initial experiences with major hepatectomies in infants with large liver neoplasms at our center.

**Materials and methods:**

From December 2023 to May 2024, four major hepatectomies were performed in pediatric patients aged 5 to 11 months. Indications, perioperative data, and surgical outcomes were retrospectively analyzed.

**Results:**

The outcomes of the four patients in this case series demonstrate the feasibility and safety of major hepatectomies in infants with large liver neoplasms. Among the cases, three involved hepatoblastoma (PRETEXT stages 2–4), and one was an anastomosing hemangioma. Radical resection (R0) was achieved in all cases, with average intraoperative blood loss 120 ml, and only one patient requiring a blood transfusion. Postoperative complications were minimal, with two cases of mild to moderate post-hepatectomy liver insufficiency (Clavien-Dindo grades 1–2) and one bile leak (Clavien-Dindo grade 2) that resolved spontaneously. No perioperative mortality or tumor recurrence was observed during a six-month follow-up.

**Conclusion:**

These results highlight the success of careful surgical planning, advanced techniques, and comprehensive perioperative management in achieving favorable outcomes for this high-risk patient population.

## Introduction

Radical treatment of patients with various types of liver tumors often requires extensive liver resection, which remains a clinical challenge, particularly in pediatric practice (1). Liver tumors in infants are rare. According to data from the American Academy of Pediatrics, 375 liver tumors in children and infants were identified over ten years of observations, of which 252 were malignant and 123 were benign (2). The most common malignant tumor was hepatoblastoma, followed by hepatocellular carcinoma in terms of detection frequency. Complete tumor removal was achieved in just under half of the children with hepatoblastoma or hepatocellular carcinoma (3). Some unresectable tumors respond well to treatment through liver transplantation, yielding favorable outcomes (4). However, in certain centers, resection is performed even in patients with high surgical risks (5).

The limited experience with liver resections in pediatric practice has resulted in a shortage of surgeons proficient in performing these complex procedures. Additionally, anatomical resections present significant challenges, requiring a comprehensive understanding of anatomical variations, particularly in vascular and biliary structures (1, 2).

In our center, only minor atypical resections of the 2nd and 3rd segments of the liver were performed until the end of 2023. Starting in 2023, to prepare for liver transplantation, our center conducted the first successful attempt at an anatomical resection of the right lobe with the involvement of surgeons experienced in liver resection and liver transplantation (6, 7). This manuscript was prepared following the CARE guidelines (https://www.care-statement.org).

## Aim

This study aimed to describe the initial experiences with major hepatectomies in infants with large liver neoplasms at our center.

## Materials and methods

The study was approved by the local ethics committee (IRB Statement # 01-1-109/408; 09.09.2024). The parents of the patients provided written consent, allowing the use of medical data for scientific research while ensuring the anonymity of the patients. The data of four patients from our center who underwent major liver resections were retrospectively analyzed.

For the patients, baseline variables such as age, sex, body weight, and date of surgery were analyzed. The PRETEXT-POSTEXT classification was used for staging hepatoblastomas (8). To assess liver failure, laboratory data including AST, ALT, total bilirubin, and INR were monitored on postoperative days 1, 3, and 5. Postoperative complications were evaluated using the Clavien-Dindo classification (9), and for patients with complications, the Comprehensive Complication Index (CCI) was calculated to assess the severity of each case (10). Long-term outcomes of surgical treatment were also evaluated. Continuous variables are presented as medians and ranges, while categorical variables are expressed as numbers and percentages.

## Results

From December 2023 to May 2024, four children aged 5 to 11 months (mean age 7.3 months) were hospitalized in our center with large neoplasms of the liver. The perioperative characteristics of the patients are presented in Table 1.

**Table 1:** Perioperative characteristics of patients.

Characteristic	Patient 1	Patient 2	Patient 3	Patient 4
Age, months	5	7	10	11
Gender, male/female	Male	Male	Male	Female
Body weight, kg	6.6	7.6	7.8	7.9
Diagnosis	Anastomosing hemangioma	Hepatoblastoma PRETEXT 3, POSTEXT 3	Hepatoblastoma PRETEXT 2, POSTEXT 2	Hepatoblastoma PRETEXT 4, POSTEXT 3
Preoperative chemotherapy, protocol, courses	Not performed	SIOPEL-3, 7 blocks	SIOPEL-3, 4 blocks	SIOPEL-3, 7 blocks
Resection volume	RL	ERL	RL	ERL
Operation time, minutes	175	255	185	195
Estimated blood loss	50	200	150	80
Need for transfusion, ml	0	100	0	50
ALT on post-op day 1, U/L	185	261	145	341
ALT on post-op day 3, U/L	89	182	76	115
ALT on post-op day 5, U/L	32	99	36	53
AST on post-op day 1, U/L	385	752	218	511
AST on post-op day 3, U/L	110	516	110	210
AST on post-op day 5, U/L	57	127	43	64
Bilirubin on post-op day 1, mmol/L	131	43	99	110
Bilirubin on post-op day 3, mmol/L	57	212	42	73
Bilirubin on post-op day 5, mmol/L	24	57	21	19
INR on post-op day 1	1.42	2.6	1.3	1.3
INR on post-op day 3	1.25	2.6	1.2	1.2
INR on post-op day 5	1.1	1.7	1.1	1.1
Complications, Clavien-Dindo	Post-hepatectomy liver insufficiency, Grade 1	Post-hepatectomy liver insufficiency, Grade 2	None	Bile leak, Grade 2
ССI	8.7	20.9	0	20.9
Postoperative chemotherapy, protocol, courses	Not performed	SIOPEL-3, 4 blocks	SIOPEL-3, 1 blocks	SIOPEL-3, 2 blocks
LOS, days	7	10	5	6

RL, right lobe; ERL, extended right lobe; ALT, alanine aminotransferase; AST, aspartate aminotransferase; INR, international normalized ratio; CCI, comprehensive complication index; LOS, length of stay.

### Case No. 1

A 5-month-old male infant, weighing 6.6 kilograms, initially presented with rotavirus infection and was hospitalized at a local healthcare facility. During her stay, clinicians noted abdominal distension localized to the right hypochondrium. Ultrasonography revealed a suspected liver tumor measuring 5.3 × 5.6 cm. One month after discharge, the child was referred to our center for further evaluation. The patient had no other medical history. Ultrasound and CT with intravenous bolus contrast revealed a tumor measuring 7.5 × 6 × 4.5 cm located in segments V-VIII of the right liver lobe. CT indicated that the tumor was heterogeneous, hypervascularized, well-defined, and progressively filled with contrast in the arterial and portal phases, without signs of contrast washout. The liver parenchyma showed normal characteristics with no signs of infiltration, fibrosis, or cirrhosis. No additional neoplasms in the liver or other organs were detected, and the lymph nodes were not affected. Laboratory data showed normal liver function, prothrombin time, international normalized ratio, and platelet levels. The alpha-fetoprotein level was 35 IU/ml. Given the CT findings, differential diagnosis included angiosarcoma and hepatoblastoma. Percutaneous needle biopsy of the tumor suggested anastomosing hemangioma. Immunohistochemical analysis revealed a positive reaction for CD-34 staining, confirming the diagnosis of anastomosing hemangioma (11). Considering the progressive growth of the tumor over the past month, the patient underwent right hepatectomy (removal of segments V, VI, VII, and VIII of the liver).

### Case No. 2

A 7-month-old male infant, weighing 7.6 kilograms, was admitted to our center for evaluation. According to the medical history, progressive abdominal enlargement had been observed since the age of 6 months. On palpation, the liver was markedly enlarged and exhibited a firm consistency. Ultrasound and CT with intravenous bolus contrast revealed a tumor measuring 10 × 8.4 × 5.5 cm, located in segments VI–VIII of the liver. CT showed the tumor to be heterogeneous, hypervascularized, without clear boundaries and contours, progressively filling in the arterial and portal phases. No contrast washout was observed. The unaffected liver parenchyma displayed normal characteristics without signs of infiltration, fibrosis, or cirrhosis. No additional neoplasms in the liver or other organs were detected, and the lymph nodes were not affected. Laboratory data indicated that the patient's platelet levels, prothrombin time, international normalized ratio, and liver function tests were normal. The alpha-fetoprotein (AFP) level was 12,500 IU/ml. The preliminary diagnosis was hepatoblastoma of the right liver lobe with extension to the fourth segment.

Percutaneous needle biopsy with immunohistochemical study confirmed the diagnosis. The patient was diagnosed with PRETEXT 3 hepatoblastoma without distant metastasis. The patient underwent 7 courses of chemotherapy according to the SIOPEL-3 protocol (carboplatin 100 mg, doxorubicin 12 mg, cisplatin 12 mg). The AFP level decreased to 5,150 IU/ml. However, control CT with intravenous contrast did not show significant tumor size reduction (POST-TEXT 3). Considering the tumor size and its central location, an anatomical extended right hepatectomy was performed, followed by subsequent courses of chemotherapy.

### Case No. 3

Another child was referred to our center for evaluation due to delayed physical development. However, following our center's standard protocol for developmental delay, an abdominal ultrasound was performed, revealing a mass in the right lobe of the liver. A subsequent contrast-enhanced computed tomography (CT) scan identified a tumor measuring 5.7 × 5.4 × 5.5 cm, located in liver segments V, VII, and VIII. The tumor was heterogeneous and hypervascularized, with well-defined boundaries and contours. It exhibited progressive enhancement during the arterial and portal phases, without evidence of contrast washout. The unaffected liver parenchyma appeared normal, with no signs of infiltration, fibrosis, or cirrhosis. No additional neoplasms or lymph node involvement were detected.

Laboratory evaluations showed normal platelet counts, prothrombin time, international normalized ratio, and liver function tests. AFP levels were elevated at 1,100 IU/ml. Differential diagnosis included hepatocellular carcinoma and hepatoblastoma. Needle biopsy and immunohistochemical analysis confirmed the diagnosis of hepatoblastoma. The patient was staged with PRETEXT 2 hepatoblastoma without distant metastasis.

The treatment plan involved four cycles of chemotherapy following the SIOPEL-3 protocol (carboplatin 100 mg, doxorubicin 12 mg, cisplatin 12 mg). Follow-up CT revealed a reduction in tumor size to 4.1 × 4.2 × 4.5 cm, with POST-TEXT 2 staging. AFP levels decreased to 120 IU/ml. However, the tumor remained centrally located, in close proximity to the inferior vena cava and middle hepatic vein, involving segments V, VII, and VIII of the liver. Considering the tumor's size, location, and characteristics, a right hepatectomy was performed. Postoperative management included additional courses of chemotherapy to complete the treatment protocol.

### Case No. 4

A seven-month-old child presented to our center with abdominal distension and severe pain. The patient had no other medical history. Imaging studies, including ultrasound and contrast-enhanced computed tomography (CT), revealed a tumor measuring 18.5 × 9.9 × 8.7 cm, located in liver segments III–VIII. The CT findings indicated a heterogeneous, hypervascularized mass with poorly defined margins and irregular contours, exhibiting progressive arterial and portal phase enhancement without evidence of contrast washout. The unaffected liver parenchyma appeared normal, with no signs of infiltration, fibrosis, or cirrhosis. No additional neoplasms were identified in the liver or other organs, and the lymph nodes were unremarkable. Laboratory investigations showed normal platelet counts, prothrombin time, international normalized ratio, and liver function tests. Alpha-fetoprotein (AFP) levels were elevated at 23,000 IU/ml.

**Figure 1:**
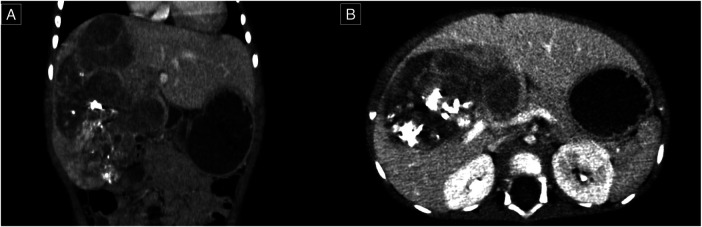
Contrast-enhanced computed tomography, venous phase. **(A)** Coronal plane; a lesion of the right lobe of the liver extending to segments VI, V, VI, VII, and VIII. **(B)** Axial plan; the lesion appears close to the portal vein and its right and left branches confluence.

A percutaneous biopsy confirmed the diagnosis of hepatoblastoma. The patient was staged as having PRETEXT 4 hepatoblastoma without evidence of distant metastasis. The treatment protocol included seven cycles of chemotherapy following the SIOPEL-3 regimen (carboplatin 100 mg, doxorubicin 12 mg, cisplatin 12 mg). Follow-up contrast-enhanced CT revealed a reduction in tumor size to 13.3 × 8.7 × 7.5 cm, corresponding to a POST-TEXT 3 stage, along with functional hypertrophy of the left lateral sector of the liver (Figure 1). AFP levels decreased to 3,320 IU/ml. Due to the tumor's size and central location, an anatomical extended right hepatectomy was performed. The patient, who was 11 months old at the time of surgery, continued postoperative chemotherapy as per the treatment protocol.

## Results

All patients underwent comprehensive preoperative evaluations for differential diagnosis, which included ultrasound diagnostics, computed tomography (CT) with intravenous bolus contrast, magnetic resonance imaging (MRI), and positron emission tomography (PET) when indicated. Additionally, standard clinical and biochemical blood tests, as well as coagulation profiles, were performed. Alpha-fetoprotein levels were assessed. If necessary, further consultations with specialized clinicians (gastroenterologists, cardiologists, urologists) were conducted.

**Figure 2:**
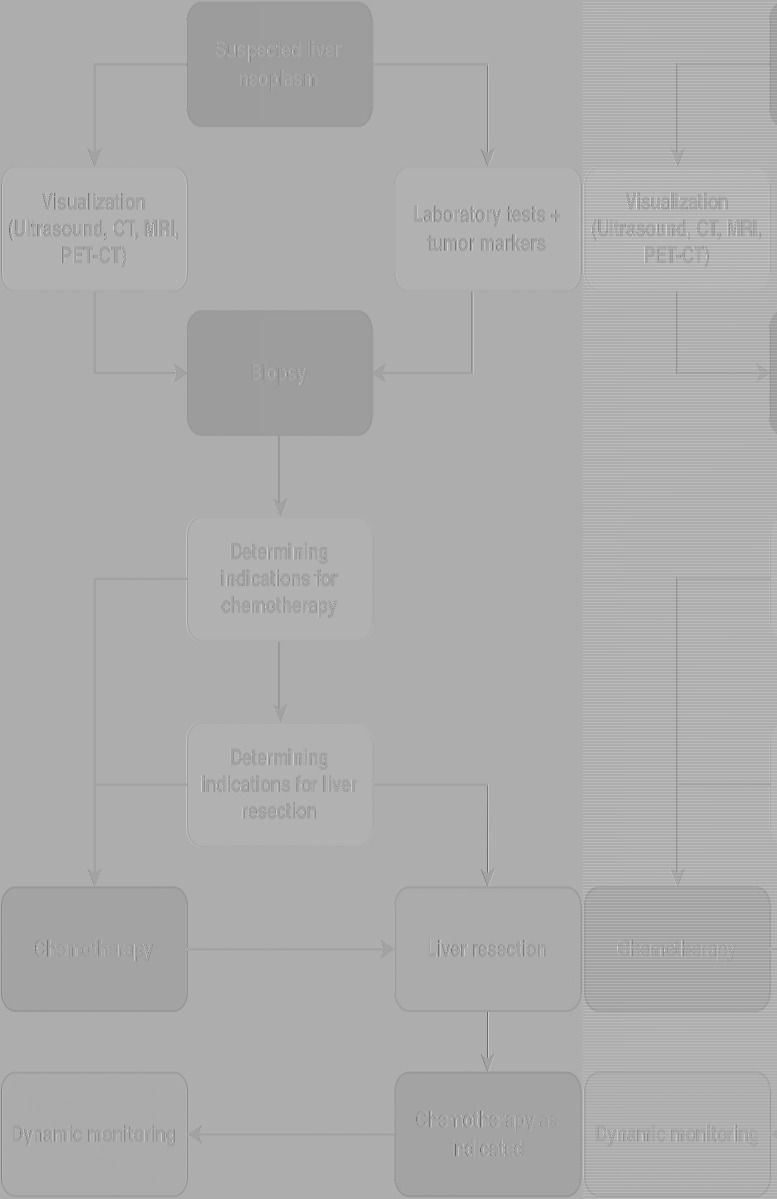
Algorithm for managing patients with liver tumors at the national Children's medical center. CT, computed tomography; MRI, magnetic resonance imaging; PET-CT, positron emission tomography-computed tomography.

All patients underwent liver biopsy prior to surgery. The diagnosis was established based on histological and immunohistochemical studies. Indications for chemotherapy were determined based on the tumor's size and differentiation. Following chemotherapy, a control CT was performed. The extent of liver resection was determined based on the tumor volume and its relationship to major and hepatic vessels. The patient evaluation algorithm is presented in Figure 2.

### Surgical technique

All patients underwent laparotomy via a bisubcostal incision. Liver mobilization was achieved by dividing the falciform, coronary, right, and left triangular ligaments. Caval gates were exposed to access the hepatic veins, followed by mobilization of the inferior vena cava (IVC) from the right lobe. Cholecystectomy was performed to facilitate visualization of the Rex-Cantlie line. The hepatoduodenal ligament was subsequently mobilized to access the afferent vessels of the right hepatic lobe. The right branch of the portal vein and the right hepatic artery were identified and ligated. In cases of extended right-sided hepatic resections, arterial and portal branches supplying segment 4 were also isolated and ligated. The confluence of the right and left bile ducts was visualized, and the resection line was marked.

**Figure 3:**
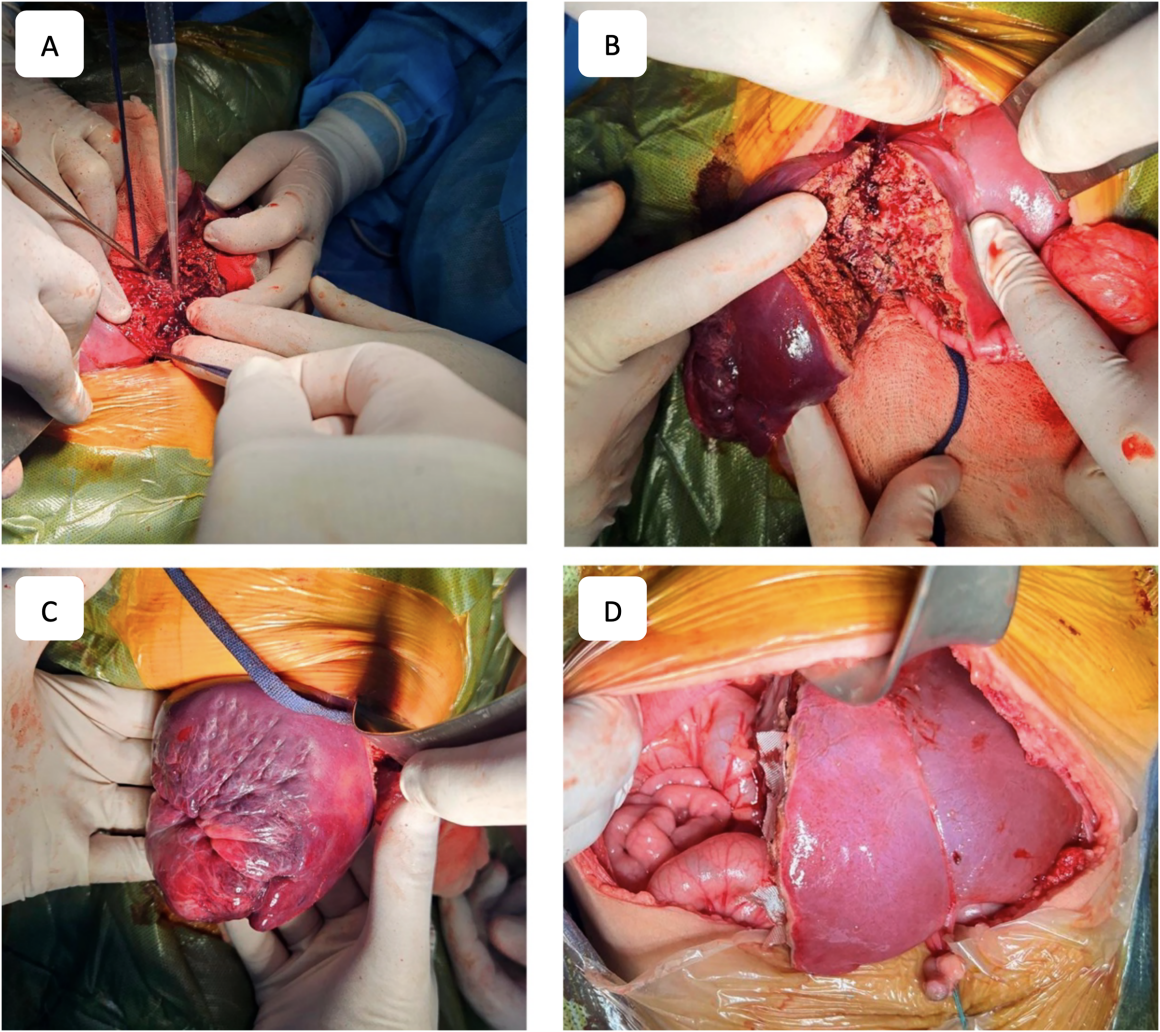
Surgical stages. **(A)** Process of liver parenchyma dissection. **(B)** Resection plane. **(C)** Right lobe of the liver after parenchyma dissection. **(D)** Final view after right-sided hepatectomy.

To ensure procedural safety and avoid IVC injury during parenchymal transection, the Hanging Maneuver technique was employed. This involved suspending the parenchyma with a tape passed beneath the right hepatic vein, the IVC, and the liver hilum. Parenchymal transection was performed using an ultrasonic dissector (Misonix SonaStar, Misonix, USA) in combination with bipolar coagulation, with continuous saline irrigation of the resection surface. Intraparenchymal vascular structures were ligated, clipped, and transected as needed. Upon completing the resection, the portal plate, containing bile ducts and vascular structures, was isolated, ligated, clipped, and transected, with additional biliostasis and suturing performed as required. The right hepatic vein was ligated and transected, and in cases of extended right-sided resection, both the right and middle hepatic veins were transected. The right hepatic lobe was subsequently removed (Figure 3).

Hemostasis was meticulously achieved using electrocoagulation and hemostatic applicators, and biliostasis was verified. The falciform ligament was restored to prevent kinking of afferent vessels to the left hepatic lobe. A drain was placed near the liver bed, and the abdominal cavity was closed. Resected liver specimens were submitted for histological and immunohistochemical analysis. In all cases, the pathological findings confirmed the preoperative diagnosis, and radical resection (R0) was achieved.

### Postoperative rehabilitation and outcomes

Following surgery, all patients were transferred to the intensive care unit (ICU) for postoperative management. Planned extubation was successfully performed within 6 h of transfer from the operating room. Continuous monitoring of vital signs was conducted, with acid-base balance and arterial blood gas levels assessed every 6 h. Key laboratory parameters were evaluated daily to ensure comprehensive postoperative care.

Patients received prophylactic antibacterial therapy, gastroprotective agents, and supportive treatments, including human albumin (20%) and fresh frozen plasma, based on clinical indications. Pain management protocols were implemented as required. Early mobilization and enteral nutrition were initiated promptly to promote recovery and minimize postoperative complications.

Moderate post-hepatectomy liver insufficiency (Clavien-Dindo grade 1–2) developed in two patients, characterized by hyperbilirubinemia, increased INR, and decreased albumin levels (see Table 1). These patients received additional albumin and fresh frozen plasma transfusions. One patient required a blood transfusion following extended right hepatic lobe resection (patient #2). Liver function parameters normalized within two days for the patient who underwent anatomical resection of the right hepatic lobe. Patient No2 with extended right hepatectomy required continued transfusion therapy (albumin, FFP) until the fifth postoperative day in a separate unit. Drain tubes were removed on the third day postoperatively for all patients, with no surgical complications observed. One patient after extended right hepatectomy (Patient 4) developed bile leakage on the second day after surgery. This leakage resolved spontaneously within 5 days postoperatively. This complication was classified as Clavien-Dindo stage II. The patient was discharged on the 6th day after surgery.

Patients undergoing resection for hepatoblastoma received postoperative chemotherapy courses (Table 1). Postoperative follow-up lasted 6 months. The first outpatient follow-up was conducted one month after surgery. Blood tests (clinical, biochemical, coagulation profile, and AFP for hepatoblastoma patients) and abdominal ultrasound were performed for all patients. Control contrast-enhanced CT scans were conducted 3–6 months postoperatively to assess primary disease recurrence, with no instances of recurrence observed in any case. AFP levels in hepatoblastoma patients were within normal range.

## Discussion

The most serious complication during liver resections in children is bleeding, both intraoperatively and postoperatively (12, 13). Unlike adults, children have a smaller circulating blood volume, where even minor blood loss can have significant consequences. Additionally, the proximity of anatomical structures in children contributes to increased bleeding compared to adult patients (14). Precise surgical technique plays a crucial role in minimizing blood loss. It is important to avoid damaging small venous tributaries draining into the inferior vena cava, which should be ligated and clipped rather than coagulated to prevent profuse bleeding (12).

Studies have shown that therapeutic reduction of intraoperative central venous pressure to 2–4 mmHg can reduce blood loss during liver resection (15). In our cases, we aimed to maintain central venous pressure below 5 mmHg to decrease blood loss and the need for blood transfusion. In the event of inferior vena cava injury, anesthesiologists were advised to intentionally elevate end-expiratory pressure to prevent air embolism.

The Pediatric Liver Tumor Study Group of the Pediatric Oncology Society conducted a prospective study (SIOPEL-1) in 2002. Among 100 patients undergoing liver resection, blood loss of less than 500 ml was observed in 60% of patients, while 13% experienced blood loss exceeding 1 liter (16). In our series of operations, intraoperative blood loss was less than 300 ml in all cases. According to Busweiler et al. (17) 45% of 73 patients undergoing liver resection required blood transfusion, whereas in our mini-series, only 1 patient received a blood transfusion.

Various methods of vascular control exist. The Pringle maneuver is often used due to its simplicity and effectiveness. However, it can lead to ischemia in the remaining liver and intestinal congestion. Complete vascular exclusion, where both inflow to the liver and the inferior vena cava are occluded, provides a bloodless surgical field but carries significant risks and may lead to patient hemodynamic instability (18). In our series, we employed selective vascular isolation, ligating only the vessels of the resected liver segment. This method prevents portal and arterial bleeding during anatomical liver resection while avoiding occlusion of the inferior vena cava. Additionally, to ensure safe resection, we utilized the “hanging maneuver” to suspend the liver over the inferior vena cava, thereby minimizing the risk of its injury.

Furthermore, careful parenchymal dissection is crucial during resection. We advocate for the use of cavitation ultrasonic dissectors, which allow for safe resection without damaging major venous vessels draining various liver segments, thereby reducing blood loss during parenchymal division (19, 20).

Despite advancements in surgical techniques, liver resection remains a complex and high-risk procedure, particularly in infants (18). There is limited literature available on perioperative and short-term outcomes in this age group. The first prospective study by the Pediatric Liver Tumor Study Group (SIOPEL-1) reported 18% surgical complications and 5% surgical mortality (16). Attempting to define short-term outcomes in children, Zwintscher and colleagues analyzed data from 126 children who underwent liver resection for malignant liver tumors in a single year, revealing 30.7% surgical complications and 3.7% mortality (21). In 2016, an analysis of postoperative complications in children undergoing liver resection for hepatoblastoma found complications in 58% of 73 patients, with no instances of early mortality within 30 days post-operation (19). In our series, we did not observe surgical complications or mortality. However, two patients experienced post-hepatectomy liver insufficiency, which was managed conservatively. The average Comprehensive Complication Index (CCI) in our series was 9.8 points. There were no observed long-term complications or recurrence of primary disease during the follow-up period.

The uniqueness of our case series lies in demonstrating the feasibility and safety of major liver resections in infants, a population with inherently higher surgical risks due to limited physiological reserves and anatomical challenges. Unlike previously reported studies, which often highlight significant intraoperative blood loss and complication rates (16, 17), our series achieved exceptional outcomes with blood loss consistently below 200 ml, only one patient requiring transfusion, and no perioperative mortality or severe surgical complications. This was accomplished through meticulous surgical planning, the use of advanced techniques such as selective vascular isolation and the “hanging maneuver,” and close interdisciplinary management. While global studies report complication rates as high as 58% and mortality up to 5% in pediatric liver resections (17, 21), our approach yielded a low Comprehensive Complication Index (CCI) of 9.8, with all patients recovering without long-term complications or disease recurrence during the follow-up period. These results underscore the importance of developing regional expertise in hepatobiliary surgery and the urgent need for improved screening and early diagnosis of pediatric liver tumors, particularly in regions like Uzbekistan where the pediatric liver tumor burden is significant. A limitation of our study is the small number of patients and the relatively short follow-up period of 6 months post-operatively.

## Conclusion

In conclusion, our case series demonstrates that with meticulous surgical techniques, interdisciplinary perioperative management, and advanced tools, major liver resections in infants can be performed safely and effectively, even in complex cases. Our results, characterized by minimal blood loss, low complication rates, and no perioperative mortality, highlight the feasibility of achieving optimal outcomes in this high-risk population. These findings emphasize the critical need for expertise in pediatric hepatobiliary surgery and the importance of establishing robust screening and diagnostic protocols for early detection and management of pediatric liver tumors, particularly in regions with a significant disease burden, such as Uzbekistan.

## Data Availability

The original contributions presented in the study are included in the article/Supplementary Material, further inquiries can be directed to the corresponding author.
